# NDP52 and its emerging role in pathogenesis

**DOI:** 10.1038/s41419-025-07668-z

**Published:** 2025-05-03

**Authors:** Krenare Bruqi, Flavie Strappazzon

**Affiliations:** Univ Lyon, Univ Lyon 1, CNRS, INSERM, Physiopathologie et Génétique du Neurone et du muscle, UMR5261, U1315, Institut Neuromyogène, Lyon, France

**Keywords:** Autophagy, Cell biology

## Abstract

Autophagy is a pro-survival process that regulates the degradation and renewal of cellular components, making it a crucial mechanism for cellular homeostasis. There are selective forms of autophagy that are specific to a number of substrates, such as pathogens (bacteria or viruses), protein aggregates or excess/damaged organelles. These processes involve as key players autophagy receptors, that link the cargo to be degraded to the autophagic machinery. Among them, NDP52 (also known as CALCOCO2) has been described to act as a “bridge” between the autophagy machinery and (1) damaged mitochondria in the mitophagy process; (2) pathogens during xenophagy or (3) proteins in the process of aggrephagy. The aim of this review is to summarize the major functions of NDP52, and to highlight the existence of two human NDP52 variants that have been described as risk or protective factors for Crohn’s disease or Multiple Sclerosis and Alzheimer’s disease patients, respectively. As these three diseases share common pathological features that lead to inflammation, such as mitochondria or gut microbiota dysfunctions, but also pathogenic infections, it seems clear that NDP52 could be a key player at the crossroad by acting indirectly on inflammation, and therefore a potential target for clinical applications and benefits.

## Facts

- NDP52 is a selective autophagy receptor implicated in mitophagy, xenophagy, and aggrephagy.
- The nuclear pool of NDP52 plays a role in gene transcription regulation and potentially in chromatin structure and organization.
- NDP52 is involved in the negative regulation of the classical NF-κB signaling pathway.
- Up to date, two variants of NDP52 have been characterized in human pathologies: a risky variant in Crohn’s disease-the NDP52^Val248Ala^, and a protective variant in Multiple Sclerosis and Alzheimer’s disease- the NDP52^G140E^.
- Crohn’s disease, Multiple Sclerosis and Alzheimer’s disease share common pathological features that lead to inflammation.

## Open Questions

- Are there other NDP52 variants implicated in human diseases besides the ones known to date?
- What role is the nuclear pool of NDP52^Val248Ala^ and NDP52^G140E^ variants playing?

## Introduction

Autophagy is an evolutionary conserved and tightly regulated catabolic process of cellular content removal through the lysosomal route of degradation [1, 2]. This process is an orchestrated interplay between several proteins and is of pivotal importance in maintaining overall cell homeostasis and function. With respect to the delivery mode of the cytosolic material to the lysosomal lumen, autophagy has been categorized into microautophagy, chaperone-mediated autophagy (CMA), and macroautophagy [1]. Microautophagy is conserved from yeast to mammals and requires the invagination of the lysosomal membrane to degrade cytosolic content [3]. In addition to microautophagy, proteins of the cytosol can reach the lysosomal compartment in a process known as chaperone-mediated autophagy. The latter mediates only the degradation of proteins that hold a pentapeptide sequence (KFERQ). They are recognized by the heat shock 70 (Hsp70) protein in a selective manner and are subsequently driven to the lysosome where they bind the lysosomal-associated protein 2 (LAMP2) [3]. Instead, macroautophagy (hereafter called autophagy), represents the most studied autophagic pathway, and consists in a series of steps during which the material to be degraded is engulfed into a double-layered membrane termed autophagosome, that ultimately fuses with the lysosome where degradation and recycling take place [1]. Deregulation of autophagy is present in many human diseases, such as neurodegenerative disorders and cancers, making it extremely well-studied [1, 4]. The removal of cellular content can either be general or highly selective. When the process is selective, the molecular mechanisms involve a set of soluble or membrane-bound receptors known as selective autophagy receptors (SARs), that recognize the cargo (i.e.: protein aggregates, organelles, pathogens). The interactions between cargo-bound receptors and ATG8 family proteins (i.e. LC3 or GABARAP proteins) anchored to the membrane of the forming autophagosome (phagophore) are crucial for the selective sequestration of the content into the latter [5, 6]. Microtubule-associated proteins 1A/1B (LC3) exist as three homologous isoforms in humans: LC3A, LC3B, and LC3C. The effective docking of the receptor-cargo complex to the phagophore depends on the LIR (LC3-Interacting Motif) interactions. One of the most well-established autophagy receptors in mammals is the Nuclear Dot Protein 52/Calcium binding and coiled-coil domain 2 (NDP52/CALCOCO2), which is encoded by the chromosome 17 and consists of 446 amino acid residues.

It was first identified in the nucleus as a component of nuclear dots-multiprotein compartments that respond to stressors, such as viral infections [7]. Later studies showed its high distribution in the cytoplasmic space [8], where it takes part in important autophagic events [9, 10]. NDP52 belongs to the group of ubiquitin-binding autophagy receptors (of soluble nature). As mentioned above, it acts as a bridging adapter in the process of selective autophagy, coupling the cargo to the autophagic machinery, from where the cargo is subsequently driven to lysosomes for degradation [5]. The selective forms of cellular content removal that implicate NDP52 are mitophagy- the process that ensures the degradation of damaged or unwanted mitochondria [11]; xenophagy- the removal of pathogenic organisms (bacteria, virus) [12–14], and aggrephagy- the degradation of protein aggregates [15]. In addition, evidence suggests that NDP52 could play a role in the selective clearance of adapter proteins, in a type of autophagy termed adaptophagy by the authors [16]. Interestingly, specific polymorphisms of NDP52 have been identified in human pathologies, highlighting the importance of this autophagic receptor in several diseases such as Crohn’s disease (CD) [17], Multiple Sclerosis (MS) [18], and Alzheimer’s Disease (AD) [19]. In this review, we have described these three pathologies and underlined the fact that they are all inflammatory diseases, linked to pathogenic infections, deregulation of the microbiota but also to the accumulation of the Tau protein (defined as primary or secondary tauopathies). Herein, we have highlighted NDP52 as a common potential target for disorders associated with inflammation and Tau accumulation.

## NDP52: structure and functions

**Fig. 1:**
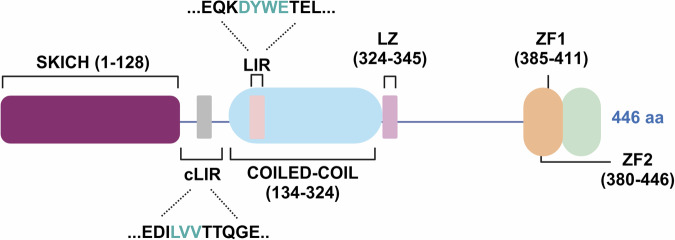
Schematic representation of NDP52 structure depicting its domains. The SKICH domain is illustrated at the N-terminal part of the protein, followed by an LC3C-interacting region (cLIR, in blue), a Coiled-coil domain comprising an LC3-interacting region (LIR, in blue), a Leucine zipper domain (LZ), Zinc finger domain 1 (ZF1) and 2 (ZF2) at the C-terminal part.

Structurally, NDP52 comprises a skeletal muscle and kidney-enriched inositol phosphatase (SKIP) carboxyl homology (SKICH) domain which facilitates its membrane localization; an LC3C-interacting region (cLIR), a coiled-coil (CC) domain that contains an LC3-interacting region (LIR), a leucine-zipper (LZ) domain, and two zinc finger (ZF) domains at the C-terminal - ZF1 and ZF2 [20, 21]. The binding of the ubiquitinated cargo occurs through its ubiquitin-binding domain (UBD), whilst linking to the autophagic machinery is mediated thanks to the interaction of the receptor with the isoform C of LC3 (LC3C) [22] that resides on the autophagosomal membrane (Fig. 1). It should be noted that in addition to bringing the cargo into autophagosomes, NDP52 is involved in the maturation of these double-layered membranes via its interaction with LC3A, LC3B, and/or GABARAPL2 through an LC3-interacting region that is distinct of the cLIR, and with MYOSIN VI (MYOVI) [13].

Additionally, NDP52 exerts its function through interacting with numerous other proteins including the nucleosome assembly protein (NAP1) [12, 23], RB1 inducible coiled-coil 1 (RB1CC1) [24], Tank-binding kinase 1 (TBK1) [12, 23], Similar to NAP1 TBK1 adapter (SINTBAD) [12], Focal adhesion kinase family interacting protein of 200 kD (FIP200) and UNC51-like kinase 1 (ULK1) [25].

## Functions of NDP52

### Xenophagy and NDP52

Infectious agents whose lysosomal degradation is mediated by NDP52 include *Salmonella enterica serovar Typhimurium (hereafter, S. typhimurium), Listeria monocytogenes, Mycobacterium tuberculosis and Shigella* [9, 12, 26]. Recognition of pathogens is done by an NDP52 ZF- containing region that can selectively recognize ubiquitin-coated bacteria [12, 26], and a galectin-interacting region (GIR) that specifically binds to the sugar receptor galectin [27]. In parallel to anti-bacterial autophagy, NDP52 is involved in innate immune response against viruses. For instance, during infection with influenza virus, viral protein PB1-F2 induces immune cell apoptosis and enhances inflammation. It was found that this protein interacts with NDP52 to regulate innate immunity by activating NF-κB and type I interferon-associated pathways in a TNF receptor-associated factor 6 (TRAF6)- dependent manner [28]. Other studies have reported that NDP52 plays a role in the viral cycle of Adenovirus type 5 (Ad5) and Herpes simplex viral (HSV-1) infection, and that interferon treatment improves its localization to the cytoplasm [7]. Furthermore, NDP52 has been defined as a sensor during infection with hepatitis B virus (HBV). In particular, by associating with the HBV envelope proteins and Rab9, NDP52 triggers a degradation process that ultimately leads to HBV clearance through the Rab9-dependent lysosomal degradation pathway. Besides elucidating the protective role of NDP52 against HBV, this work indicates that this receptor could mediate its function in lysosomal degradation routes other than through canonical autophagy [29]. Not only does NDP52 play a protective role against viral infections, but it is also effective in certain conditions for promoting virus replication [30, 31]. For instance, tripartite motif 26 (TRIM26), a member of the TRIP family of proteins, mediates the degradation of the mitochondrial antiviral signaling protein (MAVS) through NDP52 selective autophagy during pseudorabies virus (PRV) infection, which in turn favors PRV production [32]. Overall, further studies are needed to better understand the impact of NDP52-mediated autophagy in viral diseases, which could undoubtedly facilitate the designing of potential targeted strategies to counteract them.

### “Adaptophagy” and NDP52

NDP52 is involved in the negative regulation of the classical NF-κB signaling [16], whose activation is a major driver of chronic inflammation in diseases. Indeed, Inomata and colleagues revealed an interesting role of NDP52-mediated selective autophagy in Toll-like receptor (TLR) signaling, the family of receptors that trigger various innate immune responses associated with physiological and pathological immunity. In this context, NDP52 was shown to mediate selective degradation of the adapter molecule TIR domain-containing adapter inducing interferon β (TRIF) and of the TRAF6 upon poly (I:C) stimulation [16], preventing excess immune signaling that often generates a toxic level of reactive oxygen species (ROS) [33]. This work indicates that this receptor is involved in “adaptophagy”—the selective removal of adaptor proteins-crucial players in signal transduction [34]. As such, the impact of NDP52 in negatively regulating the activity of the widely known transcription factor of inflammation, suggests its link with inflammatory diseases. In line with this, NDP52 has been reported to regulate innate immunity responses and suppress canonical NF-κB activation through associating with the linear ubiquitin assembly complex (LUBAC). Findings show that the crosstalk between the two contributes to several cellular responses, including NF-κB-mediated inflammation, apoptosis, and xenophagy regulation [35]. Additionally, the correlation between NDP52 and immune response has been described in corneal inflammation in a study by Han et al. NDP52 down-regulation in primary corneal epithelial cells significantly increased the expression of pro-inflammatory genes and of TNF receptor-associated factor 2 (TRAF2), a positive regulatory protein of the NF-κB pathway, indicating that NDP52-dependent autophagy in myeloid macrophages and corneal epithelium has an anti-inflammatory effect [36].

### Mitophagy and NDP52

NDP52 is recognized as an indispensable receptor of the main common mitophagy pathway governed by PINK1 (PTEN induced putative kinase 1) and Parkin [11]. Upon mitochondria depolarization, PINK1, a protein kinase, accumulates and stabilizes on the outer mitochondrial membrane (OMM), where it phosphorylates its downstream targets including Parkin E3 ubiquitin ligase and its substrate Ub at residue Serine65. As a result, Parkin translocates to the damaged mitochondria and mediates the ubiquitination of its protein substrates on the mitochondrial surface. This activates a chain of molecular events ultimately resulting with the sequestration and engulfment of the damaged organelle into an autophagosome. Ubiquitinated mitochondria are then recognized by SARs such as NDP52, which through a cascade of protein-protein interactions attract the autophagy-initiation machinery comprising LC3, FIP200, autophagy-related 13 (ATG13), autophagy-related 16 (ATG16) [10, 25].

In this context, NDP52 is considered a redox-sensitive receptor, capable of shifting into its oligomerized state, facilitating the recruitment of these autophagic proteins around the damaged organelle for the initiation of the autophagic process. In fact, NDP52 redox-sensing capacity is due to the presence of four Cysteine residues (C18, C153, C163, C321), that upon oxidative stress are capable of forming disulfide-linked conjugates, therefore mediating NDP52 oligomerization [37]. In addition, NDP52 can exert its function in autophagy by interplaying with other proteins such as TBK1 and Ras-related protein RAB7 in cardiomyocytes, in order to promote the clearance of ischemia-induced impaired mitochondria and prevent cardiomyocyte apoptosis in acute myocardial infraction [38].

### Aggrephagy and NDP52

Protein homeostasis or proteostasis is an equilibrium that is sustained by the protein quality control network to preserve the correct folding of proteins and prevent their accumulation, which in turn could have cytotoxic outcomes [39]. A pivotal role in this process highly relies on the activity of SARs that in a selective manner recognize the ubiquitinated protein cargo and mediate their lysosomal clearance [40]. Of note, this event can occur in a ubiquitin-independent manner as well [41–43]. The main SARs involved in ubiquitin-dependent cargo recognition comprise the neighbor of BRCA1 gene (NBR1), Tollip, Sequestosome1 (SQSTM1/p62), and Tax 1 binding protein 1 (TAX1BP1) [42, 44–46]. The list grows further with NDP52, thanks to the works that have elucidated its role in this selective form of autophagy. For instance, Biel et al. have shown that NDP52 associates with p53-positive protein aggregates in breast cancer cells, and rat breast tumors to promote their lysosomal removal [15]. Whilst, with regards to neurodegenerative disorders, NDP52 implication has been demonstrated in the context of AD—a disorder well characterized by the presence of protein aggregates [47, 48]. This point has been further elaborated below.

### Nuclear roles for NDP52

**Fig. 2:**
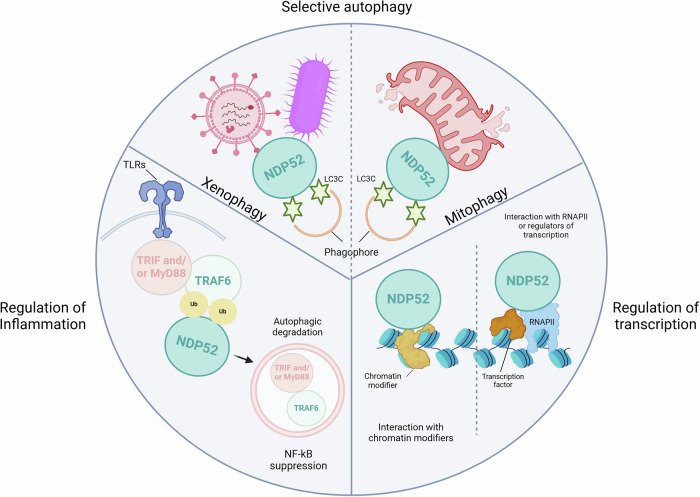
Schematic representation of the known functions of NDP52 in mammals. NDP52 is an autophagy receptor involved in selective autophagy and more specifically in the recognition and degradation of pathogens (such as bacteria and viruses) and damaged mitochondria. It is also known to regulate NF-κB signaling downstream of Toll-like receptor (TLR) pathways. Finally, NDP52 has a nuclear function as it is able to cluster with RNA polymerase II (RNAPII) at transcription initiation sites and bind double-stranded DNA to induce changes in DNA structure.

Having been initially discovered in the nucleus, the role of NDP52 within the central organelle of the cell was in shadow until 2023, but Dos Santos et al. by applying a multidisciplinary approach, characterized the biochemical properties and nuclear roles of this receptor. Authors demonstrated that NDP52 clusters with RNA polymerase II (RNAP II) within the nucleus and binds specifically and with high affinity to double-stranded DNA (dsDNA) [20]. These data provide invaluable evidence for NDP52 as transcriptional regulator, with potential functions in chromatin organization and structure. Taken together, these findings highlight NDP52 as a multifaceted protein with critical roles in several cellular pathways (Fig. 2).

## NDP52 functions in immune cells

Autophagy represents a cell-autonomous defense mechanism against pathogenic threads. The connection of autophagy and autophagy-related proteins to the response of immune cells towards diverse insults has been elaborated in previous reviews [49–51]. With regards to immune cells, NDP52 has been shown to be expressed in B lymphocytes, and to a lower extent in monocytes and T cells, in a healthy donor sample [18]. Given this, it could be interesting to investigate in the future if NDP52 expression is differentially regulated in other immune cells under pathological conditions, and in particular in disorders that feature chronic inflammation. Additionally, it would be of interest to provide a deeper understanding of the role this receptor has in B cells itself, and its potential implication in B cell function and innate immune response. Another type of immune cells that express NDP52 expression are the microglia- the guardians of the central nervous system (CNS). The study by Kim et al. conducted in an AD mouse model (APPsw/PS1dE9) has reported that the microglia that surround amyloid β (Aβ) plaques express NDP52 in their perinuclear region [48]. How NDP52 expression is regulated in microglia under normal and pathological states remains to be elucidated. Besides this, by exploring whether the autophagic activity of NDP52 is further enhancing microglia response to threads could provide new insights about their potential interplay in maintaining neuronal homeostasis and function.

Although further research is required to expand our knowledge on the molecular mechanisms via which NDP52 operates in immune cells, and pathogenesis of inflammatory disorders, a previous work has shown that NDP52-mediated autophagy of MAVS attenuates interferon signaling in a Tetherin-dependent manner.

Tetherin is an interferon-induced membrane protein that inhibits the release of viral particles from infected cells. Upon RNA virus infection, Tetherin is upregulated, and in turn recruits to the mitochondria the March8 E3 ligase to mediate Lysine 7 ubiquitination of MAVS, promoting the interaction of the latter with NDP52 and subsequently its removal through autophagy. These findings elucidate a mode of action in which NDP52 contributes to avoiding activation of excessive and potentially harmful immune responses [52].

## NDP52 in Crohn’s disease

Crohn’s disease (CD) is a chronic inflammatory disease of the digestive tract [53]. It causes inflammation and thickening of the lining, as well as ulcers and, occasionally, fissures and perforations. These lesions can affect the entire digestive tract, from the esophagus to the rectum. CD progresses in flare-ups, causing stomach pains and diarrhea that can persist for several weeks. Periods of remission between outbreaks may last a few weeks, months or even years. It is difficult to predict the frequency of attacks. An estimated 3.1 million adults (1.3%) in the United States have been diagnosed with inflammatory bowel disease (IBD), which includes CD. The exact cause of CD remains unclear, but genetic analyses have revealed that 140 loci carry genetic risk variants predispose to CD. Through this analysis, a link between susceptibility to CD and autophagy was discovered, since several autophagic genes such as ATG16L1, the leucine-rich repeat kinase 2 (LRRK2), immunity-related GTPase family M protein (IRGM) and ULK1 were defined as risk factors [54–57]. Moreover, in 2013, Ellinghaus et al. identified two novel genes as genetic risk factors for celiac disease: among the loci identified one locus harbors a low-frequency missense mutation in the gene encoding for the autophagy receptor NDP52 [17]. This NDP52 variant has a Val to Ala substitution at position 248, a mutation that is found in the ubiquitin-binding domain of the protein, and that consequently impacts its structure. Since NDP52 is known to be able to attenuate excessive NF-κB signaling [16], through functional analyses, the authors sought to elucidate the role of the variant in modulating pro-inflammatory signaling. After stimulating HeLa cells with poly(I:C)—a Toll-like-receptor 3 (TLR3) agonist, they found that overexpression of wild-type NDP52 (Val248), but not the mutant Ala248 allele impaired NF-κB activation. The authors suggested that signaling adaptors downstream of TLRs could represent selective targets for a particular type of selective autophagy that they call “adaptophagy”. Furthermore, they discovered that whereas the wild-type NDP52 was destabilized and degraded following activation of TLR3 signaling (like a receptor of “adaptophagy”), the mutant variant was more stable. They hypothesized that the region surrounding the amino acid 248 contains several Lysine residues that may represent ubiquitination targets, highlighting a potential mechanism of NDP52 turnover [17, 58]. Altogether, this study was an important discovery in the context of CD pathology, as excessive NF-κB activation is known to be one of the main drivers of chronic inflammation. This work placed NDP52 as a regulator of inflammation in CD, by interfering with the proinflammatory signaling through its noncanonical autophagy.

### CD treatments

This disorder currently remains incurable due to lack of treatment. However, besides surgery, there are several medications that find application in achieving disease control and alleviation of symptoms. For many years, corticosteroids were the main line of remedy, but due to numerous unwanted side effects, risk of mortality [59–61], and ineffectiveness in maintaining remission [62–64], their use has been limited and has been replaced by safer therapies. Currently, the standard care for CD patients consists of diverse agents including, 5-amino salicylic compounds [65], antibiotics [66], thiopurines [67], immunosuppressive therapies [68], immunomodulators [69], and a class of drugs that target proteins made by the immune system and that are known as biologics [70]. Indeed, it was the introduction of these medications that led to a paradigm shift in the management of CD, and reduced the risk of surgery [71]. The list of biologics applied in CD management consists of anti-TNF alpha, anti-integrin α4β7, and interleukin (IL) 12/23 inhibitors [72]. In addition to these medications, an emerging group of orally administered small-molecule drugs that hold promise for CD treatment are the Janus-associated kinase 1 (JAK1) inhibitors. These agents operate by attenuating multiple cytokine-related inflammatory pathways, leading to an improvement of the condition. Currently, there are three Jak inhibitors licensed for IBD treatment, but only one of them has approval for CD [73]. Besides many benefits, the use of above-mentioned therapeutics comes with a set of drawbacks, and there is plenty of space for improvement. Currently, several clinical trials are ongoing for novel treatment strategies for moderate-to-severe stages of CD, including trials for different monoclonal antibodies [74–76], JAK1 inhibitors, the potential use of fecal transplantation [77, 78], and mesenchymal adult stem cells strategies [79, 80].

## NDP52 in Multiple sclerosis

Multiple sclerosis (MS) is a very debilitating chronic autoimmune disease [81]. Depending on the region of the world, the incidence is 50 to 300 per 100,000 individuals. MS generally affects young adults with a prevalence of three female patients for every male patient. Currently, the processes that trigger MS are not known, but genetic predispositions, environmental agents and social components may participate in pathogenesis. MS exists in three different forms: (1) a cyclic or remitting form (relapsing-remitting MS, RR-MS), which affects 80–85% of patients without progression of the handicap between relapses; (2) this MS can progress to secondary progressive MS (or MS-SP); and (3) a primary progressive form (or PP-MS) in which the phases of the disease are not interspersed with phases of remission, affecting approximately 15% of patients. In all forms, the disease attacks myelin, the tissue that surrounds nerve fibers [82]. It is called multiple sclerosis because certain brain areas are affected in zones or plaques.

MS pathology is an inflammatory disease since abnormally activated lymphocytes enter the brain and attack myelin sheaths. Inflammation therefore leads to demyelination, causing axon damage and neuron degeneration, resulting in disability [83]. Among other hypothesized mechanisms that explain the diffusion of neurodegeneration present in MS patients is the involvement of mitochondrial dysfunctions [84]. Mitochondria are essential intracellular organelles involved in ATP synthesis, calcium regulation and when damaged, they are major sources of ROS and of mitochondrial DNA (mtDNA). The release of mtDNA from mitochondria is in turn capable of promoting inflammation [85]. Consequently, mitochondrial dysfunction in the pathological processes of MS leads to inadequate energy production, intracellular dysregulation and inflammation that worsens the disease. It has been demonstrated that autophagy-related 5 (ATG5) and Parkin increase in the cerebrospinal fluid and serum of MS patients, highlighting these autophagy/mitophagy elements as molecular markers of the disease’s active phase [86, 87]. Moreover, in 2021, Di Rita et al. identified and characterized a variant of the autophagic receptor NDP52 that directly links mitophagy and MS pathogenesis [18]. Indeed, by sequencing the DNA coming from peripheral blood mononuclear cells (PBMCs) of 203 RR-MS patients with specific probes for the genes encoding for two main mitophagic receptors, NDP52 and Optineurin, the study revealed a significant association of NDP52 c.491G>A mutation (rs550510, p.G140E) with a decreased susceptibility to MS, suggesting that the NDP52 variant NDP52^G140E^ may confer protection to MS. Since mitochondrial dysfunctions are related to MS pathogenesis and since NDP52 serves as a bridge between mitochondria and phagophores during degradation of damaged mitochondria by autophagy, the role played by NDP52^G140E^ in mitophagy was assessed. Structural and biochemical analysis indicated that the NDP52^G140E^ variant exhibits a better affinity for the LC3C protein. It seems that the single amino acid substitution in the NDP52 sequence is crucial for extending its noncanonical LIR motif (cLIR) and promoting its binding with LC3C, thus favoring a more efficient clearance of damaged mitochondria with respect to the wild-type (WT) form. Additionally, this study highlights that, among PBMCs, NDP52 is mainly expressed in the B cell subpopulation ensuring effective mitophagy following B cell stimulation and reducing the release of the proinflammatory cytokine tumor necrosis factor alpha (TNF-α). Of note, because enhanced immune response may benefit MS patients by eliminating microbes, and because NDP52 negatively regulates TLR-triggered NF-κB activation, the NDP52^G140E^ variant protective effect in MS was also tested on the activation of NF-κB, following TLR stimulation [18].

However, the data obtained indicate that the G140E substitution in NDP52 does not affect its function in regulating TLR signaling. In conclusion, this work leads to propose a model in which the variant G140E of NDP52 may prevent the release of pro-inflammatory cytokines from B cells (and maybe mitochondrial factors such as mtDNA) known to trigger inflammation in MS patients.

### MS treatments

Although MS still remains an incurable disease, thanks to extensive research, the therapeutic armamentarium for managing it has remarkably expanded during the last decades. Currently, the approach to reducing biological activity is based on the use of disease-modifying therapies (DMT). This class of drugs is able to impact the course of the disease by suppressing or modulating immune function [88]. Given that MS represents a complex disorder, currently available medications have been designed with the aim of improving certain aspects of it. The list of frequently applied DMTs for relapsing form of MS includes Ocrelizumab- a humanized monoclonal antibody against CD20 molecule of the B cell surface [89], Natalizumab- an α4β1 integrin inhibitor [90], Fingolimod- a S1P inhibitor that blocks infiltration of autoreactive lymphocytes into the CNS [91], dimethyl-fumarate [92], Teriflunomide [93], interferon beta (IFN-β) [94], glatiramer acetate [95]. Currently, secondary-progressive forms of MS are treated with Siponimod-a selective S1P modulator, Ocrelizumab, Cladribine and diroximel fumarate [96]. Instead, the only approved drug for the treatment of primary-progressive MS is Ocrelizumab [97]. Several other candidate therapies and therapeutical approaches are undergoing clinical trials. For instance, given the increasing evidence that human herpes virus and human endogenous viruses are linked to MS etiology and pathogenesis, antiviral drugs are being tested for potential benefits [98]. An increasing interest is also oriented towards novel treatment strategies that could stimulate re-myelination including Retinoid X receptor-γ agonists, anti-Leucine-rich repeat and Immunoglobulin-like domain-containing protein 1 (LINGO1) antibody, Olexosime, mesenchymal stem cells, Wnt signaling pathway modifiers and other agents such as progesterone [99].

## NDP52 in Alzheimer’s disease

Alzheimer’s disease (AD), the common cause of dementia in elder people is a progressive neurodegenerative disorder whose pathological hallmarks are the extracellular deposition of Aβ plaques and the intracellular accumulation of neurofibrillary tangles (NFTs) in the brain [100]. The latter are formed as a consequence of aberrantly phosphorylated Tau (pTau) protein, ultimately causing neuronal dysfunction and death. Given the presence of Tau aggregates, AD classifies as tauopathy alongside other diseases such as frontotemporal dementia linked to chromosome-17, progressive supranuclear palsy, corticobasal degeneration, Pick’s disease, Niemann-Pick disease type C, and chronic traumatic encephalopathy [101].

Of note, the attempts in generating therapeutics against AD are partially oriented in the designing of strategies that would promote the efficient removal of pathologically phosphorylated Tau. In healthy neurons, autophagy is the primary route of Tau clearance [102]. However, in AD neurons evidence suggests that autophagy is compromised, subsequently leading to the progressive accumulation of protein aggregates [103, 104]. To date, pTau has been reported to colocalize with a few autophagic receptors [105], including NDP52 [47, 48]. In particular, it has been found that in an AD mouse model (APPsw/PS1dE9), NDP52 associates with pTau in the cortex and hippocampal regions of the brain [48]. This interaction between Tau and NDP52 requires the SKICH domain of the receptor [47]. Intriguingly, in addition to pTau, NDP52 was shown capable of associating with intracellular Aβ as well [48]. However, further investigations are required to elucidate the link between the two. In addition, Jo et al. reported that the activation of the nuclear factor erythroid-2 (Nrf2) axis, a well-defined defense mechanism against oxidative stress, reduces pTau levels by inducing NDP52 expression in primary cortical neurons and in CN1.4 mouse cortical cells. The evidence about the direct implication of NDP52 in pTau removal was reinforced by co-immunoprecipitation experiments on cerebral cortex tissues from AD patients. On these samples, NDP52 was associated with pTau and their levels were inversely correlated, hinting that likely this receptor, by promoting pTau clearance, might in turn prevent Tau aggregation [47]. In this regard, a recent article by Mattioni et al. reported that NDP52 favors degradation of pathological Tau in in vitro systems but also in vivo in a *Drosophila melanogaster* model of Tau-induced AD. Most interestingly, the NDP52^G140E^ variant of NDP52 described previously in the context of MS, emerged to be presumably a genetically protective factor for AD.

**Fig. 3:**
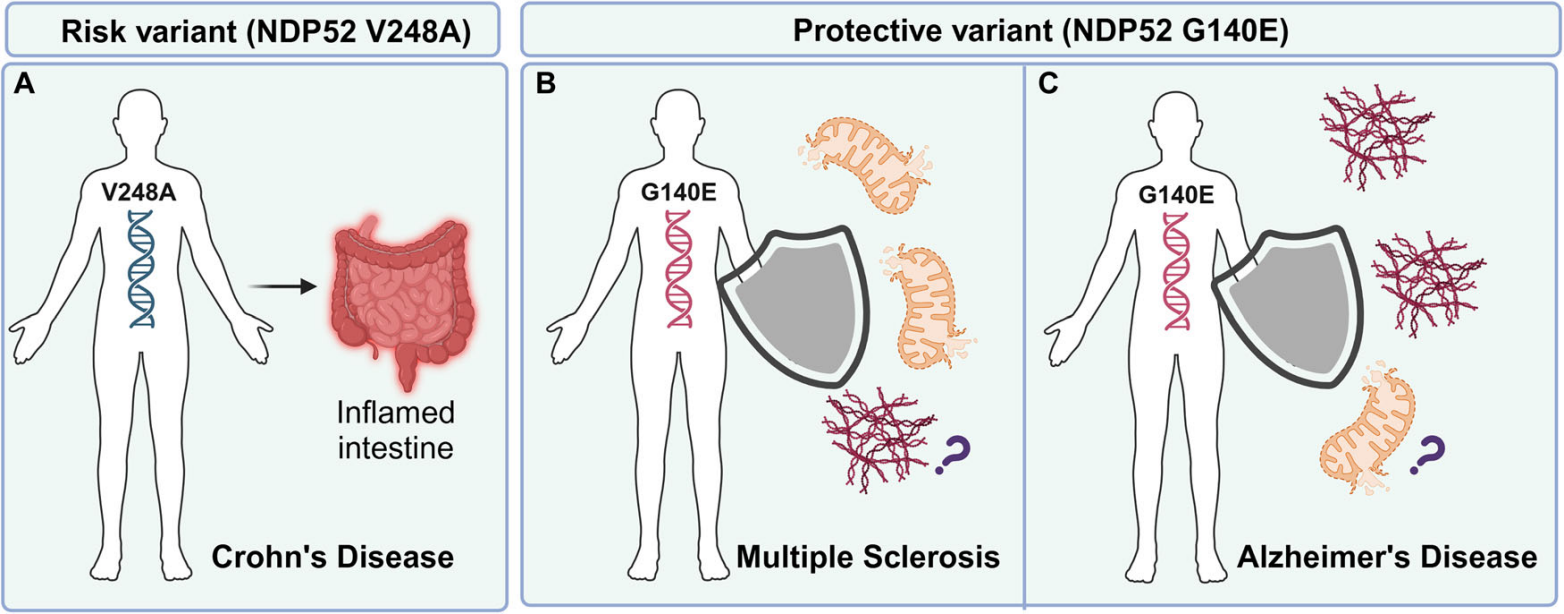
Effects of two human variants of NDP52 in diseases. **A** NDP52^V248A^ mutant is a risk factor for patients affected by Crohn’s disease. Under TLR activation, it remains more stable than its WT form and favors inflammation by boosting the NF-κB signaling. **B**, **C** NDP52^G140E^ variant is a protective factor for patients affected by Multiple Sclerosis and Alzheimer’s Disease. It acts by favoring the autophagic clearance of damaged mitochondria and the autophagic clearance of pathological forms of Tau.

In particular, Mattioni et al. found that the NDP52^G140E^ variant is much more effective than NDP52^WT^ in reducing the accumulation of pathological forms of Tau via the autophagic process, and in rescuing the typical neurodegenerative phenotypes induced by hTau toxicity in *Drosophila melanogaster*. Mechanistically, NDP52^WT^ and NDP52^G140E^ bind phospho-Tau with comparable efficiency, but NDP52^G140E^ binds the autophagic machinery (LC3C and LC3B) more efficiently than its WT form, which could explain its greater efficiency in phospho-Tau removal [19]. Altogether, this work underlines the NDP52^G140E^ variant as a beneficial factor for AD patients (Fig. 3). In accordance with these data, Tumurbaatar et al. have shown that preserved autophagy in humans, and at least an autophagy pathway mediated by NDP52, may help in the maintenance of cognitive integrity in non-demented individuals with Alzheimer’s neuropathology (NDAN). These individuals are known to remain cognitively intact while presenting neuropathological features such as Aβ plaques and NFTs. In fact, this study performed in *post-mortem* brain samples from age-matched controls, AD and NDAN subjects, demonstrated that among a number of autophagy-related proteins evaluated, significantly lower levels of NDP52 were found in AD patients compared to control condition, whilst interestingly, in NDAN subjects, NDP52 was significantly elevated with respect to AD cases [106]. Of note, a standing-out feature of the neuropathology in NDAN individuals compared to AD is the absence of Aβ oligomers, and decreased Tau oligomers at hippocampal synapses [106]. As such, these findings suggest that reduced Tau pathology as a result of the NDP52-mediated autophagy through removal of Tau oligomers might be protecting cognitive integrity. Interestingly, Jo et al. found no correlation between the amount of phosphorylated Tau and the amount of NDP52 in soluble fractions from AD patient cortex. By contrast, the amount of Tau, as well as phosphorylated Tau (12E8 and PHF1) in insoluble sarkosyl fractions was found to be inversely proportional to that of human NDP52 [47]. In agreement with Mattioni et al. [19], this work indicates that NDP52 may prevent Tau protein aggregation by the autophagic clearance of phosphorylated forms of Tau.

A striking feature of assembled Tau is its ability to transfer between cells and seed the aggregation of monomeric soluble Tau [107]. This process is believed to stand behind the amplification and propagation of Tau inclusions in the AD brain [108]. In this context, autophagy mediated by NDP52 has been shown to play a protective role as a defense mechanism against seeded Tau aggregation in vitro. Indeed, the entry of Tau assemblies into the cytosol gets sensed by galectin 8-known as ‘the danger’ receptor, which consecutively activates autophagy by recruiting NDP52. The latter links to the galectin-8-associated cargo through its galectin-8 binding domain, preventing the incursion of Tau seeds into the cytosol [109].

Altogether, the presented findings highlight the implication of NDP52 in AD, by not only promoting the autophagic removal of pTau, but also as well hindering Tau spreading, thus potentially preventing aggravation of AD pathology. In line with this, it should be thus interesting to check in the future whether the NDP52^G140E^ variant may act also on Tau spreading. Even though many aspects of AD remain elusive, it is plausible that this disorder is favored by chronic neuroinflammation. It has been proposed that mitochondrial dysfunctions and microglia-driven neuroinflammation are key factors that sustain Tau pathological development and spreading [110, 111]. Mitochondria, as the main hub of ROS synthesis, play an important part in this regard. In AD, the accumulation of malfunctioning mitochondria due to mitophagy impairment leads to an increase in oxidative stress, mtDNA release, microglial activation and consequently neuroinflammatory response [112]. Currently, there is a knowledge gap regarding the role NDP52 holds in this context, and additional investigations are required. A summary of the effects of NDP52 variants in the contexts of CD, MS and AD is illustrated in (Fig. 3).

It should be noted that alongside NDP52, p62 is another well-known autophagic receptor for Tau protein. A better understanding of the interplay these receptors have with monomeric and pathogenic forms of Tau will undoubtedly facilitate the designing of potential therapies to counteract the accumulation of protein aggregates in AD. With regards to this, Ferrari et al. have recently demonstrated that pathogenic forms of Tau are capable of evading autophagic clearance [113]. In particular, they have shown that ubiquitinylated Tau fibrils from AD patients’ brains are sequestered into larger condensates by the oligomeric p62. The condensation reaction is mediated by the NBR1 receptor. Intriguingly, these p62:NBR1-coated Tau fibrils fail to recruit TAX1BP1-the main upstream attractor of the autophagic machinery, due to the masking of fibril ubiquitin moieties by p62. Additionally, these fibrils are resistant to deubiquitylation, consequently making their interaction with the p62 irreversible.

Altogether, this study sheds light on a mechanism that impedes the proper p62-mediated autophagic clearance of Tau fibrils in the AD brain and provides insights into potential future approaches that could artificially promote their removal, by for example tethering the pathogenic cargo to TAXBP1 instead of p62 [113]. Taken together, presented evidence puts autophagy under the spotlight as a crucial process in cell homeostasis and function. Although further research is needed for a more profound comprehension, reported findings support the statement that autophagy has got a protective role against Tau pathology, highly encouraging the designing of autophagy-inducing therapies as a potential future strategy of AD treatment.

### AD treatments

According to Alzheimer’s disease international (ADI), in 2020 the total number of people suffering from dementia was around 55 million. This number is estimated to double every 20 years, reaching 78 million in 2030 and 139 million in 2050. Such predictions are an emergency call to our society to further deepen our understanding of AD and improve treatment outcomes. Currently approved pharmacological strategies include the administration of drugs that act on neurotransmitter disbalance in the brain. Such agents belong to two families: acetyl cholinesterase inhibitors (Donepezil, Galantamine, Rivastigmine), and anti-glutaminergics (Memantine). Although these medications positively impact the quality of life of AD patients, they are only symptomatic-based approaches, and their beneficial effect is limited and temporary as the disease progresses [114]. However, research evidence gathered during the last decades has provided us with invaluable insights about AD pathogenesis and progression. For instance, for many years, the Aβ cascade [115] has been at the center of investigations, and a prime target for the development of AD therapy. Following extensive efforts in this direction, nowadays we witness the application of Aducanumab and Lecanemab- the two approved monoclonal antibodies against Aβ plaques [116]. Of note, a plethora of other Aβ-targeting therapies are ongoing clinical trials [117]. Although Aβ plaques have long been under the spotlight for potentially targeted therapeutic avenues, we should note that AD is characterized by the heavy presence of NFTs composed of phosphorylated Tau aggregates. The increase in knowledge with regards to Tau biology, NFTs formation, and Tau propagation in the brain has created a strong foundation that certainly is of great help in the designing of potential therapies and disease biomarkers. To date there are no effective Tau-targeting treatments, however, the pipeline of drug candidates for tauopathies are in discovery and preclinical phases, as well as early phase trials. Potential therapeutics are dominated by immunotherapies, followed by drugs that target Tau aggregation, and to a lower extent by drugs that target Tau synthesis, Tau post-translational modifications, neuroinflammation, MAPT inhibitors and Tau clearance [118]. In addition, previous research evidence has shown promising outcomes of a proteolysis targeting chimera (PROTAC) drug. The latter is a novel small molecule named C004019 designed to simultaneously recruit E3 ligase (Vhl), and selectively enhance Tau ubiquitination, and its subsequent proteolysis. The application of this drug was able to promote Tau clearance in vitro and in an AD mouse model (3xTg-AD), leading as well to an improvement in their cognitive functions [14]. Another strategy to target intracellular protein aggregates has been reported in a study by Benn et al.

In their work, the authors exploited the cluster-dependent activation of the Triptate motif-containing protein 21 (TRIM21) in order to generate degraders combining the RING domain of this E3 ubiquitin protein ligase with a target-specific nanobody. The delivery of these degraders into a mouse model of tauopathy (Tg2541), led to a reduction in Tau pathology, suggesting that this approach can be used to reduce Tau protein aggregates rapidly and durably at scale in the mammalian brain [119]. Altogether, these findings highlight the significance of novel and efficient potential strategies that would promote Tau removal, thus preventing or attenuating the downstream cascade of events that consists of Tau assembly into oligomers and ultimately into NFTs.

## NDP52 at the crossroad between inflammation and Tauopathies

All forms of MS show a neurodegenerative process [120, 121], and the cause of progressive MS is not understood. From a clinical point of view, it emerges that, unlike RR-MS, patients with progressive MS do not respond to immunosuppression treatment as the RR-MS patients. Therefore, no effective treatment exists for them. Interestingly, some studies have shown that phosphorylated forms of Tau protein accumulate in PP-MS patients [122]. As stated above, the intracellular accumulation of misfolded or aggregates of Tau is the cause of a plethora of disorders known as “tauopathies” [123]. In tauopathies, one of the causes of disease progression is linked to the transcellular propagation of Tau seeds. Indeed, pathological assemblies of proteins that form in one cell exit to gain entry to connected neurons and thereby propagate the disorder through specific brain networks in a process termed “seeding” [124]. In 2022, LaCroix et al. demonstrated that Tau seeds are present in the brain of progressive MS patients and that these pathological Tau aggregates are found outside the MS lesions [125]. The authors suggested that MS pathology may be defined as a potential secondary tauopathy. Interestingly, CD, which is part of the inflammatory bowel disease family, has been also recently connected to Tau accumulation [126]. Altogether, presented evidence suggests that NDP52 may play an essential role between inflammation and tauopathies, however, further studies are needed to better understand this point. Taken together, NDP52 could be a common target for diseases associated with inflammation and Tau accumulation.

In analogy to what has been discovered in the context of MS and AD, a major question emerges: could NDP52^G140E^ variant play a protective role (through its action on mitochondria and/or pathological Tau) in CD or other diseases associated with inflammation and Tau protein accumulation?

Additional research is required, but we cannot therefore rule out the possibility that precision therapy strategies based on NDP52 could become crucial in the development of drugs for AD, MS, CD, and possibly other inflammatory diseases linked to Tau accumulation such as postencephalitic parkinsonism [127], or the chronic traumatic encephalopathy [128]. It should be noted that in addition to inflammation, CD, MS, and AD are three pathologies that are directly linked to pathogen infections. Indeed, a link between MS and infection with the Epstein-Barr virus (EBV) has been proved [129]. It is certain that the virus is not the only cause of MS since 90% of the population is infected with EBV during its lifetime, but it appears to be a trigger factor that has been identified in predisposed individuals. Moreover, several studies suggest that infections with EBV increase the risk of developing neurological diseases such as AD [130]. Moreover, the micro-organisms normally present in the digestive system - the intestinal microbiome are also thought to play an important role in CD and MS [131, 132]. For example, transplantation of viruses from people with IBD, and from those with healthy guts into mice have revealed interesting proof that the gut could be involved in disease pathogenesis. Mice given healthy viruses showed no changes in gut health, whilst mice given viruses from people with IBD experienced an increase in inflammation [133]. More studies are needed to understand what viruses make up a healthy human virome. Of note, the known causes of tauopathy are multiple, but they also include viral infections [134]. Interestingly, autophagy is known to be implicated in the regulation of the intestinal microbiota given that it allows the elimination of intracellular pathogens, thereby limiting the replication and dissemination of pathogenic micro-organisms. Since NDP52 is known to play key role in xenophagy, we do not exclude the possibility that, despite the findings that highlight a pro-mitophagy function of NDP52^G140E^ in MS, a beneficial effect on pathological Tau degradation in AD, this variant may also emerge as a novel link among (1) xenophagy, a form of selective autophagy of pathogens (such as bacteria and viruses) and (2) viral infections known to be associated to MS and AD (i.e., EBV, one of the main environmental risk factors for MS and AD).

Hence, future research will be required to better characterize and understand the molecular mechanisms underlying the function of both protective and risky NDP52 variants.

## Conclusion

Understanding the risk and protective factors for complex diseases such as CD, AD or MS is essential for understanding, predicting, diagnosing, and treating them. A change in the DNA sequence of the gene coding for NDP52, making a person susceptible to developing or developing CD has been described. This mutation occurs in the UBD domain (Val248Ala) of the receptor and seems to be involved in its stability under TLR stimulation. The NDP52^Val248Ala^ variant clearly favors inflammation in CD patients, through activation of the NF-κB pathway. On the contrary, the substitution located near its cLIR region (G140E), renders NDP52 more efficient in binding the autophagic machinery, and seems to be protective for MS and AD patients. However, additional studies in larger cohorts of patients are required to further strengthen current data and to better understand if this variant could be protective in other tauopathies as well. Furthermore, it is of interest to investigate whether besides effective Tau removal NDP52^GE^ acts also in the remotion of damaged mitochondria in AD. Knowing whether a person has a pathogenic or protective variant can help prevent, diagnose, and treat diseases. Even if these mutations described in NDP52 are completely different, there are some pathological features among diseases in which NDP52 is known to play a role (AD, MS and CD), such as dysfunctional mitochondria, altered gut microbiota, pathogenic infections and inflammation. It will be thus interesting in the future to better understand if NDP52 could be involved in the crosstalk between these disorders, in order to exploit potential applications and clinical benefits of NDP52.
